# COVID-19 Vaccine Administration: Phase 2 of an in Progress Review in New York State Local Health Departments

**DOI:** 10.3390/ijerph192013030

**Published:** 2022-10-11

**Authors:** Sarah Bloomstone, Molly Fleming, Mayela Arana, Emily D’Angelo, Sarah Ravenhall, Marita Murrman

**Affiliations:** 1 New York State Association of County Health Officials (NYSACHO), Albany, NY 12110, USA; 2 Region 2 Public Health Training Center (PHTC), Mailman School of Public Health, Columbia University, New York, NY 10032, USA; 3 Mailman School of Public Health, Columbia University, New York, NY 10032, USA

**Keywords:** COVID-19 response, emergency preparedness, public health systems, governmental public health workforce, vaccine administration

## Abstract

Since the onset of the COVID-19 pandemic in New York State (NYS), local health departments (LHDs) have worked to mitigate the highly infectious disease. As lead public health experts in their communities, LHDs are responsible for providing communicable disease control, emergency response, and establishing immunization programs, including leading large-scale vaccine distribution efforts. The aim of this qualitative study was to understand the processes used by LHDs in NYS to administer COVID-19 vaccines, as well as identify successes and challenges, and highlight lessons learned to improve future mass vaccination campaigns. Data were collected in two phases: (1) extant data collection of public communications; and (2) discussion groups with public health leaders across the state. Notable themes from both phases include: partnerships, programmatic elements, communication, role of LHD, State-LHD coordination, and human and physical resources. Analysis of both public and internal communications from LHDs across NYS revealed several core challenges LHDs faced during COVID-19 vaccine rollout and identified innovative solutions that LHDs used to facilitate vaccine access, administration, and uptake in their communities. Findings from this multi-phase qualitative analysis support the need to bolster the capacity and training of the local public health workforce to ensure preparedness for future public health emergencies.

## 1. Introduction

Coronavirus disease 2019 (COVID-19), first identified in December 2019, is caused by the severe acute respiratory coronavirus 2 (SARS-CoV-2) [1]. Transmitted through respiratory droplets, the virus has resulted in over 600 million cases globally, and more than 6.5 million deaths [2]. Since the onset of the pandemic, local health departments (LHDs) have played an invaluable role in the COVID-19 response across the country [3]. When the first case of COVID-19 was discovered in New York State on 1st March 2020, New York’s LHDs worked around the clock to fight the highly infectious disease and mitigate its spread in their communities. In the initial months of the pandemic, New York’s LHDs responded to the COVID-19 pandemic by: activating and mobilizing emergency preparedness plans; connecting vulnerable individuals to essential resources; informing community members; and serving as communicable disease experts by conducting case investigations, contact tracing, issuing and enforcing isolation and quarantine orders, and establishing testing clinics, all while coordinating response efforts with the New York State Department of Health (NYSDOH) and the Centers for Disease Control and Prevention (CDC) [4].

Predating the pandemic, the scope of public health and the need for robust public health infrastructure has grown. However, funding and resources have not increased in proportion with the new demands of the public health sector [3]. For example, in NYS, under Article 6 Public Health Law, LHDs are legally responsible for providing communicable disease control, emergency response, and establishing immunization programs in their communities. Despite LHDs’ collective decades of experience in emergency response and communicable disease control, budget constraints and staffing shortfalls, which have plagued LHDs for years prior to the onset of COVID-19, caused challenges in LHDs’ response efforts. State-allocated funding for LHDs decreased by more than 40% from 2011 to 2015, and LHDs outside New York City (NYC) saw a 33% reduction in their workforce from 2011 to 2017, leaving an understaffed and underfunded local public health workforce to respond to the greatest public health threat in a century [5]. LHDs across the country have had similar experiences with chronic underfunding, understaffing, and limited technical infrastructure. During the pandemic, LHDs nationwide were challenged by ambiguous or limited authority, inadequate data sharing and technology platforms, insufficient funding, and limited workforce expansion and capacity [3]. Despite these difficulties, LHDs, including across New York State, were able to fill in gaps during the initial months of COVID-19 response with creative solutions. New York’s LHDs coordinated with local social service providers to deliver wraparound services, cross-trained staff to expand their departments’ contact tracing capabilities, and shared timely and evidence-based updates–solidifying their reputation as trusted infectious disease control experts in their communities.

In December 2020, the first COVID-19 vaccine was approved by the Food and Drug Administration (FDA) under Emergency Use Authorization (EUA), marking the beginning of an extensive COVID-19 vaccine campaign across New York State and the United States. Prior to the COVID-19 pandemic, both the United States federal government and New York State government invested funding aimed towards developing a robust statewide emergency preparedness system to ensure efficient and effective responses to public health emergencies, including mass vaccination campaigns. LHDs have prepared for large-scale vaccine distribution efforts for decades and have vast expertise in emergency preparedness, both from running drills and from previous public health emergencies, such as the 2009 H1N1 outbreak, during which LHDs ran points of dispensing (PODs) that ensured access to vaccines for priority populations. While expertise and lessons learned from previous vaccination efforts were beneficial, the COVID-19 vaccine administration process was not without its challenges. Following the H1N1 outbreak, it was recommended that federal, state, territorial, tribal, and local public health departments work intentionally and collaboratively to address anticipated communication and coordination problems [6]. However, ultimately, vaccine distribution efforts, especially those related to equitable allocation of scarce resources, came down to state governments, which often faced political backlash in response to public health measures [7]. This politicization has been seen throughout the United States COVID response and globally [8].

**Figure A1:**
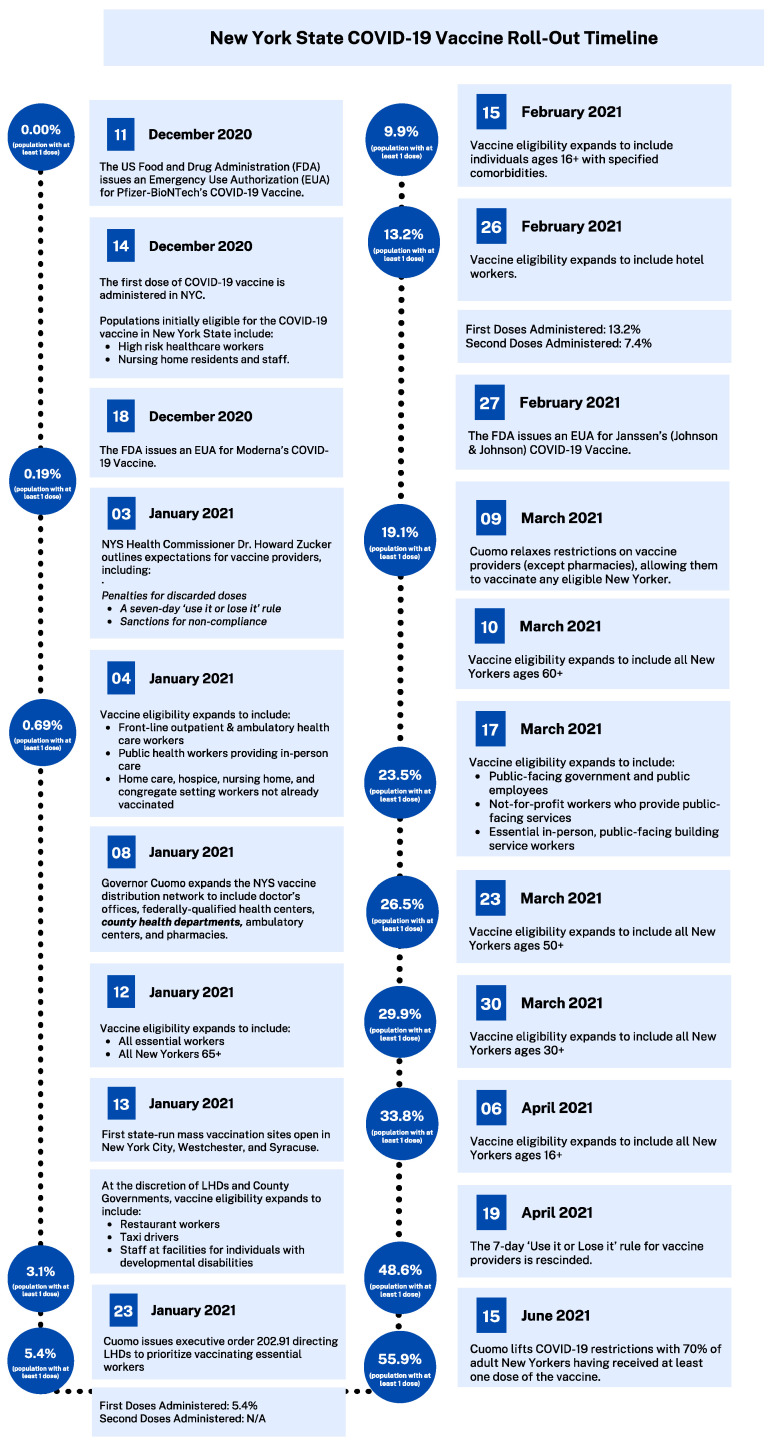
Timeline of Vaccine Distribution in New York State.

Throughout the pandemic response, the New York State Association of County Health Officials (NYSACHO), as the membership association for all 58 of New York’s LHDs, partnered with the Region 2 Public Health Training Center (R2PHTC), which provides training and technical assistance to the governmental public health workforce in New York, as well as New Jersey, Puerto Rico, and the US Virgin Islands, to collect qualitative data from LHDs to describe lessons learned and identify areas of improvement for future emergency responses in New York involving LHDs. The qualitative study described here aims to synthesize LHDs’ COVID-19 vaccine allocation and administration processes, identify successes and challenges faced, and highlight lessons learned to improve future mass vaccination campaigns. For a timeline of LHD’s vaccine administration in New York State, see Figure A1 in the Appendix A.

## 2. Materials and Methods

### 2.1. Study Design

Conducted by NYSACHO, in collaboration with the R2PHTC, this qualitative study consisted of two phases: (1) Secondary or extant data collection of publicly available online sources published between 1 December 2020 and 3 July 2021; and (2) Primary data collection in the form of an After-Action Activity taking place at the annual Public Health Leaders Summit hosted by NYSACHO in the fall of 2021. This study was approved by Columbia University Irving Medical Center’s institutional review board and all participants consented for inclusion (Protocol number AAAT0829).

### 2.2. Phase 1: Qualitative Analysis of Extant Data

Qualitative e-research, including the use of extant data (i.e., “collections of posts of text, images, media or other user-generated content”), adapts the traditions of qualitative research methods to new information and communication technologies [9,10]. At the time of data collection (summer of 2021), LHDs were still in the midst of their COVID-19 vaccine administration efforts. Collecting extant data allowed researchers to gather a rich source of data, while being sensitive to the time and capacities of county health officials.

Extant data was collected using two approaches. The first method involved reviewing all vaccine-related resources and articles linked in NYSACHO’s weekly newsletters distributed between 1 December 2020, and 3 July 2021. The second method of data collection involved a systematic audit of each LHD’s website, press releases, and social media for vaccine-related content published between 1 December 2020, and 3 July 2021. The New York City Department of Health and Mental Hygiene, which serves the 8.3 million residents of the five counties (boroughs) that comprise New York City, was excluded from this phase of analysis due to its unique governance and structure. The final dataset compiled direct quotations of vaccine-related content from identified media sources (i.e., news articles, official press releases, and social media posts) into a comprehensive Excel spreadsheet. Each quotation was also tagged with the date of publication and the corresponding county and region.

### 2.3. Phase 2: After-Action Review

In the second phase of this study, LHD health commissioners and directors participated in an After-Action Activity during NYSACHO’s 2021 Public Health Leaders Summit (henceforth referred to as “The Summit”), an annual meeting held for New York State LHD leaders. During the conference, attendees were split into groups and asked to answer questions about their departments’ most and least effective vaccination strategies; gaps and challenges they encountered during vaccine administration; future action steps for their department; and systemic changes beyond the scope of their department, needed to improve future mass vaccination campaigns and emergency response coordination. Participants were instructed to answer the questions with respect to work done under their departments or counties’ control.

The Summit was held in a hybrid format, with in-person attendees split into groups based on their Regional Economic Development Council (REDC) region, and with virtual attendees, regardless of region, participating as one group via Zoom. Some REDC regions were combined or split up to ensure each in-person group had between 3-6 participants to enable open discussion. In cases where REDC regions had to be combined, researchers worked to ensure that participants in each group came from counties with similar population sizes or geography. Additionally, while the virtual group was not split up by REDC region, similar methods were used in the initial In-Progress Review (IPR), which focused on early COVID-19 response efforts [11].

Each in-person group at the Summit had a NYSACHO board member assigned to facilitate the discussion and record responses. Following the Summit, NYSACHO staff transcribed the hand-written responses into an Excel spreadsheet for analysis. Prior to analysis, member checking of group responses by each group’s facilitator took place. The purpose of this process was to: (1) ensure the validity of the qualitative data collected by confirming that all responses were accurately transcribed and captured the LHD experience at the time of the Summit; and (2) allow for additional context to be added to clarify original responses [12]. Group facilitators were instructed not to delete or add any new information based on events experienced after the Summit.

### 2.4. Thematic Analysis

In phase 1, themes were generated inductively after closely reviewing the data in its entirety. Several iterations of line-by-line coding occurred until the thematic framework was finalized, and each quote had at least one code attached to it. The overarching purpose of this qualitative analysis was to understand the processes and challenges of vaccine distribution and administration from the perspective of LHDs throughout the state, as communicated to the public.

In phase 2, following member checking, two researchers used an iterative process of textual analysis based in grounded theory and facilitated by Dedoose software. The researchers employed line-by-line coding to identify emerging themes, and iteratively refined the codes to develop a final code structure. The final code structure was shared with NYSACHO staff for validation.

## 3. Results

### 3.1. Participants

#### 3.1.1. Phase 1

The final dataset consisted of 935 direct quotations collected from content published between 1 December 2020, and 3 July 2021. Codes were not mutually exclusive, as the same excerpt could be coded under multiple themes. All 57 LHDs included in the study were represented with at least one quotation in the final dataset.

#### 3.1.2. Phase 2

A total of 39 LHD leaders participated in the After-Action Activity, representing 38 of 58 local health departments, with 12 LHD leaders participating virtually, representing 12 counties, and 27 participating in-person, representing 27 counties. One county was represented in both the virtual and in-person groups. Overall, 67% of local health departments, excluding NYC, were represented.

The majority of participants were Public Health Directors (24), while 10 were Commissioners of Public Health, and five participants held another leadership role within their LHD (e.g., deputy director, epidemiologist, health educator). Regional participation ranged from 40% to 100%, with Central NY having the lowest participation rate and Long Island and the Mid-Hudson having the highest. Medium sized counties had the highest participation rate (70.59%), with small (65.38%) and large and extra-large counties (64.29%) having slightly lower participation rates. However, the majority of counties represented in the After-Action Activity were small (17), compared to 12 medium counties, and nine large or extra-large counties. A full breakdown of participant characteristics can be seen in Table 1.

### 3.2. Notable Themes

Thematic analysis occurred separately for phase 1 and phase 2. However, with considerable overlap across the two final coding frameworks, notable themes that emerged across both phases are presented together here. Notable themes included: partnerships, programmatic elements, communication, role of LHD, and State-LHD coordination. In addition, one notable theme, human and physical resources, was discussed almost exclusively by LHD leaders during the After-Action Activity. Notable themes are presented in Table 2.

#### 3.2.1. Partnerships

Over half of New York’s LHDs described how partnerships–both new and existing–helped in efforts to administer COVID-19 vaccines throughout their communities. Partnerships mentioned most frequently included: hospitals, pharmacies, local health care providers, educational institutions (e.g., schools and universities), community organizations, and other county-based departments (e.g., police and fire departments; local transit authorities; local Agencies on Aging; public works departments). Several LHDs, primarily during After-Action discussions, explained the impact that partnerships with their local Medical Reserve Corps (MRC) had on vaccine administration processes. One county attributed the success and efficiency of their vaccine clinics to the support of their MRC and other community volunteers. Other LHDs noted that partnerships with community volunteers and local organizations were important for enhancing equity and reducing racial/ethnic disparities in vaccination rates. During internal discussions among LHD leaders, the importance of the MRC was often framed in the context of needing additional funding to expand their staffing capacity and sustain the network of volunteers for future emergencies. Some counties also highlighted the challenges associated with creating and maintaining partnerships as an important area for improvement. For example, one LHD noted challenges associated with enrolling local health care providers as COVID-19 vaccine providers due to concerns around complex storage, handling, and administration requirements.

#### 3.2.2. Programmatic Elements

In both public-facing and internal discussions, LHDs described specific programmatic elements they utilized during vaccine distribution, including procedures for appointment registration and approaches to administer vaccines equitably throughout their communities. Due to the phased rollout of the COVID-19 vaccine, as eligibility began to expand in early 2021, LHDs had to manage high demand for the vaccine, with initially limited supply. LHDs developed multiple strategies for notifying eligible residents about upcoming vaccine appointments. Through waiting lists and stand-by lists, counties developed comprehensive censuses of eligible residents who were interested in being vaccinated. When appointments or extra vaccine doses at the end of the day became available, LHDs used these lists to notify residents. Some LHDs established systems with other county agencies (i.e., Agencies on Aging) to help older adults register for vaccination appointments over the phone or through family members, to avoid inequities associated with accessing the primarily online vaccine registration system.

Various approaches were used to administer vaccines to community members. The most common strategies employed, based on the extant data collected, were community-based vaccination sites, mobile clinics, and in-home vaccinations for homebound individuals. Common community-based pop-up sites included: churches or other faith-based organizations, local schools, community events, and other community institutions (e.g., town halls, community centers). Some LHDs partnered with community health care providers and pharmacies to facilitate vaccinations via trusted sources of healthcare information. In addition to these more common strategies, LHDs used other innovative approaches to increase accessibility and interest in vaccination. Some of these approaches included: themed vaccine clinics for eligible youth (i.e., prom-themed); drive-through vaccine clinics; and “on demand” clinics where businesses could request vaccine providers to come on-site and vaccinate eligible individuals or employees. Many of these approaches were referenced by counties and LHD leaders alike as key strategies to enhance vaccine equity, particularly among racial/ethnic minorities and other underserved communities.

LHDs’ public communications about vaccine programs were most often announcements and reminders of vaccination opportunities. In contrast, LHD leaders in the After-Action Activity described these efforts retrospectively, commenting on the successes or failures of the implementation. For example, although many LHDs publicly described targeted vaccine clinics in zip-codes with low vaccination rates, in the After-Action Activity, one LHD leader noted the limited success that such targeted efforts had on vaccine uptake.

#### 3.2.3. Communication

Throughout the COVID-19 pandemic, LHDs were responsible for communicating key public health and safety information to their communities. To this end, LHDs used a variety of public communication channels, including: social media, press releases, informational hotlines, dedicated websites, public forums, and public service announcements. A key messaging strategy utilized by LHDs on their platforms was encouraging community members to speak with trusted experts and community leaders to make decisions about vaccination. LHD leaders in the After-Action discussion commented that using trusted messengers (e.g., faith leaders, community leaders, family members, primary care physicians) was a major success in their vaccine administration efforts, helping them to share their message with harder-to-reach communities and increase vaccination rates.

During the After-Action Activity, LHD leaders had more opportunity to reflect on the challenges and successes of their communication strategies. Some LHDs described the benefits of using social media to disseminate information, provide updates, and engage communities. Others commented on how public demand for frequent COVID-19 status updates posed a challenge to LHDs’ social media communications. In addition, the prevalence of negative comments and rapid spread of misinformation via social media further complicated LHDs’ efforts to communicate with their residents. Despite these challenges, several LHDs acknowledged the importance of social media as a communication tool. Some LHD leaders even noted a need for dedicated media specialists to improve online public health messaging and engagement in the future, as well as comprehensive social media training for existing Staff.

Many LHD leaders also commented on challenges associated with communicating with the state. Due to frequently changing guidance, as new vaccines were approved or eligibility guidelines expanded, information from the state was sometimes rolled out unpredictably and with short notice, creating difficulties implementing new guidance at the local level. For example, one LHD leader noted that at times during the rollout, LHDs received important updates at the same time as the general public through executive-level press briefings, creating the need for a quick turnaround to implement new directives and provide guidance to their communities.

#### 3.2.4. Role of LHD

LHDs frequently commented on their department’s role in the vaccine administration process, particularly the importance of local decision-making for effective vaccine planning and administration procedures. The most common comment from LHDs described their prior experience in planning, practicing, and executing mass vaccination campaigns in their communities. Additionally, as the lead public health experts for their communities, several LHDs conveyed that they best understood the needs of their communities and were therefore well-positioned to administer vaccines in ways that met local needs. Despite their leadership position in their communities and years of experience in vaccine distribution, many LHDs described contextual factors related to the pandemic that challenged their ability to efficiently administer vaccines, including: lack of local input in decision making; limited vaccine supply; and staff capacity. Considering these challenges, many LHDs served as vocal advocates for their communities, encouraging the state to address these factors.

#### 3.2.5. State-LHD Coordination

One of the most common themes across both phases of data collection, related to the challenges associated with coordinating vaccine rollout between the State Department of Health and Executive Branch (henceforth referred to as “The State”) and LHDs. These concerns fit primarily into two categories: incident command system (ICS) planning and intergovernmental affairs. LHDs commented on elements of the State’s ICS planning–referring to the structure and implementation of their vaccine administration plans. Many LHDs expressed concern with the State’s initial decision to use regional hospital hubs as the primary conduits for vaccine distribution and administration, despite LHDs’ extensive experience in this role, effectively circumventing previously established LHD plans for vaccine rollout. The most common concerns related to ICS planning shared through LHDs’ public communications related to vaccine supply, allocation procedures, and provider restrictions. Especially early in the vaccine rollout, when supply was limited nationwide, LHDs commented on the allocation procedures employed by the State, particularly when large counties received a fraction of the doses they requested. Several LHDs also noted that, even when supplies were limited, the State appeared to prioritize allocations to state-run sites, rather than to local providers and LHDs. In their public-facing communications, LHDs often expressed difficulties associated with provider restrictions (i.e., that only certain providers could vaccinate certain eligibility groups) instituted early in the vaccine rollout. Many felt that these restrictions, coupled with limited vaccine supply, slowed down efforts to vaccinate communities quickly. In After-Action discussions, several LHD leaders commented that ICS planning for future emergencies must be improved to better align with the plans, experience, and capacity of local providers and LHDs.

The other main area of concern was intergovernmental affairs, which refers to interactions between state and local agencies. Most often, these comments reflected disagreements in the strategies used to regulate vaccine rollout. Several LHDs felt that vaccination efforts should have been more heavily led by medical or public health professionals to minimize the politicization of vaccination within New York State. Additionally, many LHDs felt that instituting penalties for providers who had unused doses or who vaccinated ineligible individuals was counter-productive and even hampered vaccine administration progress and uptake.

#### 3.2.6. Human and Physical Resources

In reflecting on their vaccine administration procedures, LHD leaders frequently commented on the human and physical capital needed to successfully administer vaccines on a mass scale. Discussions of these themes represent a thematic divergence between LHDs’ public communications and internal communications collected during the After-Action Activity. While some LHDs did comment on these themes in press releases or social media posts, most public communications focused on the other themes previously described.

LHD leaders described staffing as a key asset that facilitated effective vaccine administration. Nearly all discussion groups highlighted the use of various personnel types (i.e., MRC, volunteers, community organizations, first responders) as central to effective vaccination efforts. Staffing, training, and cross-training of personnel were commonly cited by LHD leaders as key areas for improvement locally and systemically. One LHD leader noted that a key systemic change to improving future emergency responses would be to cross-train all LHD staff in emergency preparedness functions.

LHD leaders also commented frequently on the physical resources needed to execute mass vaccination campaigns, including: technology, vaccine supply, storage, and public health infrastructure funding. Technology, such as GIS mapping and data management software, were recognized by several participants as key physical resources that LHDs need improved capabilities in. However, the most common physical resource that LHD leaders mentioned was funding. LHDs from nearly all regions felt that increased funding for local public health infrastructure is a critical systemic change needed to bolster future outbreak management procedures.

## 4. Discussion

### 4.1. Challenges and Solutions

Evaluating LHDs’ public communications allowed researchers to capture real-time evolutions in vaccine programs, as opposed to relying solely on retrospective commentary from the After-Action discussion groups. Analysis of both public and internal communications from LHDs across New York State revealed several core challenges and the innovative solutions that LHDs employed to facilitate vaccine access, administration, and uptake.

One key strategy LHDs used was collaboration with community stakeholders and partners, which aided LHDs in overcoming staffing challenges, reaching specific populations, and coordinating logistics throughout the vaccine roll-out. Formal cross-sector collaborations, including with local community partners, have declined over the past decade. Such partnerships proved to be essential to LHDs in New York and elsewhere for fostering engagement and trust in local public health institutions [3]. While these collaborations were successful, this reliance on volunteers highlights that LHDs are understaffed and too often lack the necessary resources, funding, and personnel needed to carry out the essential public health services they are required to provide to their communities.

LHDs had mixed experiences with social media throughout the pandemic. While they were able to expand their reach by sharing information and updates via social media, it also established an expectation for more frequent communication and created more opportunities for negative comments aimed at LHDs. As the public health workforce has worked to combat COVID-19 in their communities, they have also had to confront the rapid spread of mis- and disinformation on- and offline. While misinformation represents unintentional dissemination of misleading or false information, disinformation is intentional and can be used to undermine the public health response [13,14]. LHDs have a responsibility to communicate vital health information to their communities, and many already have risk communication plans in place [14]. For many, the rapid spread of misinformation via social media created a challenge for already overwhelmed LHDs who felt they lacked the time and resources needed to effectively refute misinformation and address community concerns. Despite these challenges, many LHDs across the state were successful in leveraging new or existing social media platforms to disseminate important information and guidance, while fostering engagement through innovative storytelling campaigns featuring local residents and trusted community leaders.

Coordination and communications with the State frequently came up as a barrier to effective vaccine rollout for LHDs. In the State’s initial COVID-19 vaccine distribution plans, LHDs were largely overlooked as critical on-the-ground partners, despite decades of experience in planning and operating mass vaccination sites. However, during weekly calls with the State, NYSACHO and LHD leadership were able to emphasize the important role and history of LHDs as vaccinators for their communities and pressed the State to prioritize LHDs to receive weekly vaccine shipments, which they began to receive in early 2021.

Additionally, with initially limited groups eligible for vaccination, NYSACHO, based on LHD expertise and input, advocated for expanding eligibility to vulnerable populations, such as migrant farm workers, homebound individuals, and other persons living in congregate settings. These examples highlight the importance of frequent communications and opportunities for collaboration between state and local health officials during an emergency to offer constructive feedback, adjust processes as needed, and ensure statewide coordination.

### 4.2. Best Practices

Based on the results of this multi-phase qualitative study, we recommend the following best practices:

- By building and maintaining relationships with a broad range of county agencies, such as Social Service, Mental Health, Information Technology, and Sheriff and Fire Departments; community organizations, such as churches, schools, businesses, and shelters; and local clinical partners, including pharmacists, hospitals, and health care providers; LHDs can increase their workforce capacity during emergencies. In addition to building these partnerships, many LHDs were successful in engaging communities, to receive vaccines for example, by collaborating with a key community leader such as a pastor or a local policymaker/elected leader to carry the public health messaging and invite their communities to an event where vaccines were being offered. Those trusted community leaders are critical in meeting concerned community members where they are at, addressing concerns, and ultimately ensuring equity in the provision of vaccines.
- LHDs can expand their operating capacity rapidly during a public health emergency through regular cross-training of health department staff and staff from other county agencies, such as Social Service and Mental Health Department employees, in basic emergency preparedness and infectious disease functions, including case investigations and contact tracing. EMS Paramedics played a critical role in vaccinating those who were homebound, presenting an additional department for cross-training in public health emergencies.
- Public health preparedness drills, including POD set-up, and operations drills enhance and expedite LHDs’ emergency response capabilities during actual emergencies.
- LHDs are highly experienced in emergency planning and vaccine distribution therefore, LHD plans for emergency response should be utilized in future mass vaccination campaigns.
- LHDs benefit from a proactive, coordinated, and multi-faceted approach to communicating with the public during a public health emergency, utilizing a variety of channels, e.g., social media, press releases, interviews, etc., to reach the widest audience possible with important public health messaging. In the future, LHDs would benefit from regular training in social media communications to enhance engagement through social media and combat misinformation.
- State and local coordination is vital to the success of mass vaccination campaigns and the State should consider and incorporate LHD perspectives and capacity through all phases of emergency response.

### 4.3. Future Recommendations

The COVID-19 pandemic made obvious that while public health responses led by skilled professionals are invaluable, the system cannot be sustained without continued investment. Many of the challenges described both in extant data collected and in After-Action groups, such as staffing constraints and lack of resources for public education, can be linked back to LHD funding deficits. An increased investment in public health by both the state and federal governments is needed for LHDs to be able to hire, train, and retain a competent workforce that will be the backbone of local public health response in future emergencies. In particular, the inclusion of emergency preparedness training and retraining for all LHD staff will help increase staff capacity during future emergencies. In addition to increasing the number of staff employed at LHDs, the mental health and wellbeing of existing public health staff must be prioritized. The stress the public health workforce has endured over the course of the COVID-19 pandemic has resulted in extreme burnout and post-traumatic stress symptoms, causing many staff to leave their positions. If left unaddressed, LHDs will continue to experience high rates of staff turnover, losing valuable institutional knowledge [15].

The COVID-19 pandemic also demonstrated the vital role LHDs play in their communities as boots-on-the-ground public health experts. LHDs are in the best position to understand their community’s unique needs, collaborate with community stakeholders, and serve as trusted sources of information for their communities. Their perspective should therefore be considered throughout all stages of an emergency response, from planning to implementation. Additionally, LHDs should be given the necessary resources to make decisions at the local level as much as possible. For example, in instances where state-level guidance is being prepared for public release, LHDs should have access to embargoed guidance in advance to prepare informational documents and resources for their communities.

To serve as a trusted and credible source of information for communities, LHDs should develop and implement a robust, coordinated communications strategy to better position themselves to serve this role in the future, and as we continue to experience the lingering effects of mis- and disinformation campaigns that have minimized trust in public health and public health institutions. Comprehensive communications training, which focuses on transparency with the public, including via social media, is needed to better enable LHDs to effectively disseminate critical health information across a variety of platforms to wide-ranging audiences. Partnerships with community organizations serving certain subsets of the population can help develop and test consistent messaging that is factual, tailored, and culturally competent. LHDs should also partner with local news outlets to help ensure dissemination of consistent and accurate information to the public [13]. Finally, it is important that LHDs continue to post and engage with community members through social media channels in order to establish these platforms as consistent and credible sources of public health information [14]. As highlighted above, to implement these recommendations, LHDs will require additional, sustained funding to ensure they have the appropriate staffing and resources to meet the communities’ information needs and get ahead of mis- and disinformation.

### 4.4. Strengths and Limitations

A key strength of this study was working directly with LHD leaders to assess their own departments’ capabilities and capacities, which highlighted a vital, but often overlooked perspective, that NYSACHO is uniquely positioned to capture and amplify. Additionally, by collecting extant data in the first phase of this study, researchers obtained real-time data on LHDs’ vaccine administration strategies and progress, while respecting local health officials’ time and capacity to participate in a study amid pandemic response. By collecting additional feedback during the After-Action Activity, researchers were able to compare this real-time data to LHD leaders’ internal reflections on the successes and challenges of the strategies they employed. LHD leaders’ perceptions do not, however, represent an objective account of LHD performance, and lessons learned are not generalizable to LHDs across the United States.

This research also highlights the benefits of partnership between practice and academia, as this study was the result of collaboration between the Health Resources and Services Administration’s R2PHTC, run through the Columbia University Mailman School of Public Health, and NYSACHO, a long-time practice partner on the training and technical assistance needs of New York State’s local public health workforce. Together, R2PHTC’s knowledge and experience in academic research and NYSACHO’s experience working with local county health officials made this study possible.

A key limitation of this study was the use of extant data. Due to this method, the dataset from phase 1 was limited to the information and experiences that LHDs published on their social media platforms, press releases, and websites. A LHD’s experience may be entirely different than the challenges described in external communications, but if that experience was not publicized, it was not included in this analysis. This limitation was balanced by the inclusion of direct qualitative data from After-Action Activity.

## 5. Conclusions

Across the initial In-Progress Review and this Vaccine Addendum, several key themes were consistent in both study phases, demonstrating the effectiveness of cross-sectoral partnerships and the need to address systemic challenges, such as intra-governmental collaboration and staffing constraints [11]. Notable strengths included the use of partnerships with county agencies and community organizations, cross-training employees to expand LHD operating capacity, and the dedication of LHD staff. Recurrent challenges included funding constraints, inequitable distribution of materials, including testing kits and associated supplies, personal protective equipment (e.g., gloves, face coverings, N95 respirators, eye protection), and vaccine administration supplies between counties, and communications with the State. As New York State moves forward and begins to look beyond the COVID-19 pandemic, serious consideration should be given to the current capacity and ongoing training needs of the local public health workforce. Challenges facing LHDs need to be addressed on a systemic level to ensure preparedness for future public health emergencies.

## Figures and Tables

**Table 1:** After-Action Activity Participant Demographics.

Variable	Count ^1^	Participation Rate Across the State ^2,3^
By Title (n = 39) ^4^		
Commissioner	10	83.33%
Director	24	53.33%
Other (Deputy Director, etc.)	5	NA ^5^
By Region (n = 38) ^6^		
Capital Region	4	50.00%
Central NY	2	40.00%
Finger Lakes	7	77.78%
Long Island	2	100.00%
Mid-Hudson	7	100.00%
Mohawk Valley	3	50.00%
North Country	5	71.43%
Southern Tier	4	50.00%
Western NY	4	80.00%
By County Size (n = 38) ^6^		
Small (<75,000)	17	65.38%
Medium (75,001–199,999)	12	70.59%
Large & Extra-Large (>200,000)	9	64.29%

^1^ Count represents the number of participants in each variable category. ^2^ Participation Rate represents the number of participants in each variable category over the total number in each variable category across New York State (e.g., 10 commissioners participated in the After-Action Activity, out of 12 total local health commissioners in New York State (excluding NYC)). ^3^ NYC was excluded from this analysis and therefore not included in participation rate calculations. ^4^ Total by title was calculated using the number of individual participants (n = 39). ^5,6^ Total by region and county size were calculated using the number of individual counties represented (n = 38).

**Table 2:** Thematic Framework of Notable Themes across Phases of Data Collection.

**Theme**
Subtheme
**Partnerships**
Partner Types (e.g., health services, county entities, public works, local government, schools or educational institutions, inter-county/regional partners, elder or senior services, social service agencies, community-based organizations, community volunteers, Medical Reserve Corps, Faith communities)
Partnership Impact
**Programmatic Elements** (Describes elements of the actual vaccine program implementation/design)
Registration & Notification Systems
Administration Approaches (e.g., “closed” clinics, community providers, community-based sites, drive-through clinics, in-home vaccinations, mobile vaccination clinics, themed clinics, vaccine ‘on-demand’)
**Communication**
Communication Methods (e.g., social media, trusted messengers, informational materials, one-on-one conversations, PSAs, online forums, student ambassadors, dedicated websites or phonelines)
Communication Challenges
Areas of Improvement
**Role of the LHD**
Prior Planning, Experience & Training
Advocacy
Local Focus (i.e., understanding of community needs; centering LHD perspective)
Contextual Challenges (i.e., vaccine supply, state decision making)
**State-LHD Coordination**
Incident Command System Planning
Intergovernmental Affairs
**Resources**
Physical Resources (e.g., technology, vaccine supply & storage, funding & infrastructure)
Human Resources (e.g., staffing, training & cross-training)

## Data Availability

Not applicable.

## References

[1] Al-Kuraishy H.M., Al-Gareeb A.I., Al-Hussaniy H.A., Al-Harcan N.A.H., Alexiou A., Batiha G.E. (2022). Neutrophil Extracellular Traps (NETs) and COVID-19: A new frontiers for therapeutic modality. Int. Immunopharmacol..

[2] World Health Organization WHO Coronavirus (COVID-19) Dashboard. https://covid19.who.int/.

[3] DeSalvo K., Hughes B., Bassett M., Benjamin G., Fraser M., Galea S., Garcia J.N., Howard J. (2021). Public Health COVID-19 Impact Assessment: Lessons Learned and Compelling Needs. National Academies of Medicine. https://nam.edu/wp-content/uploads/2021/04/Public-Health-COVID19-Impact-Assessment-Lessons-Learned-and-Compelling-Needs.pdf.

[4] New York State Association of County Health Officials (2021). New York State Local HEALTH Department Preparedness for and Response to COVID-19: An In-Progress Review. https://www.nysacho.org/wp-content/uploads/2021/03/IPR-report-FINAL.pdf.

[5] New York State Association of County Health Officials (2019). Local Health Department Budget and Staffing Trends, 2011–2017. https://www.nysacho.org/wp-content/uploads/2019/03/LHD-Budget-Trends.pdf.

[6] Fraser M.R., Blumenstock J. (2021). Lessons Relearned? H1N1, COVID-19, and Vaccination Planning. J. Public Health Manag. Pract..

[7] Tewarson H., Greene K., Fraser M.R. (2021). State Strategies for Addressing Barriers during the Early US COVID-19 Vaccination Campaign. Am. J. Public Health.

[8] Sabahelzain M.M., Hartigan-Go K., Larson H.J. (2021). The politics of COVID-19 vaccine confidence. Curr. Opin..

[9] Salmons J. (2016). Using social media in data collection. The SAGE Handbook of Social Media Research Methods.

[10] Salmons J. (2016). Doing Qualitative Research Online.

[11] Ravenhall S., Levy N.A., Simpson K., Fleming M., Arana M., DiManno P., Grijalva Y., Murrman M.K. (2021). New York State Local Health Department Preparedness for and Response to the COVID-19 Pandemic: An In-Progress Review. J. Public Health Manag. Pract..

[12] Birt L., Scott S., Cavers D., Campbell C., Walter F. (2016). Member Checking: A Tool to Enhance Trustworthiness or Merely a Nod to Validation?. Qual. Health Res..

[13] Rodgers K., Massac N. (2020). Misinformation: A Threat to the Public’s Health and the Public Health System. J. Public Health Manag. Pract..

[14] Manganello J.A., Penta S., Whalen E. (2022). Pandemic Communication: Challenges and Opportunities for Local Health Departments. J. Public Health Manag Pract..

[15] deBeaumont (2021). STAFFING UP: Workforce Levels Needed to Provide Basic Public Health Services for All Americans. https://debeaumont.org/wp-content/uploads/2021/10/Staffing-Up-FINAL.pdf.

